# 5,17-Dibromo-26,28-dihy­droxy-25,27-diprop­oxy-2,8,14,20-tetra­thia­calix[4]arene

**DOI:** 10.1107/S1600536811013043

**Published:** 2011-04-13

**Authors:** Ling-Ling Liu, Lu-Sheng Chen, Jian-Ping Ma, Dian-Shun Guo

**Affiliations:** a Department of Chemistry, Shandong Normal University, Jinan 250014, People’s Republic of China

## Abstract

In the title compound, C_30_H_26_Br_2_O_4_S_4_, the thia­calix[4]arene unit adopts a pinched cone conformation, with one of the ether-substituted rings bent towards the calix cavity and the two phenolic rings bent outwards. The phenyl rings make dihedral angles of 27.12 (9), 36.71 (10), 75.04 (8), and 76.01 (7)° with the virtual plane defined by the four bridging S atoms. The two opposite ether-substituted rings are almost parallel to each other, with an inter­planar anagle of 2.99 (12)°, while the two phenolic rings are nearly perpendicular to each other, making a dihedral angle of 74.52 (11)° and a Br⋯Br distance of 13.17 (2) Å. Two intra­molecular O—H⋯O hydrogen bonds between the OH groups and the same ether O atom stabilize the cone conformation. In the crystal, two different chains of mol­ecules, one with alternating and the other with tail-to-tail orientations, are formed by inter­molecular offset-face-to-face π–π stacking inter­actions with distances of 3.606 (3) to 4.488 (4) Å between the centroids of the aromatic rings.

## Related literature

For general background to the chemistry of thia­calix[4]arenes, see: Shokova & Kovalev (2003 ▶); Lhoták (2004 ▶); Morohashi *et al.* (2006 ▶); Kajiwara *et al.* (2007 ▶); Guo *et al.* (2007 ▶). For the synthesis and related structures, see: Lhoták *et al.* (2001 ▶); Kasyan *et al.* (2003 ▶); Desroches *et al.* (2004 ▶); Kasyan *et al.* (2006 ▶); Morohashi *et al.* (2006 ▶); Xu *et al.* (2008 ▶); Chen *et al.* (2010 ▶). For π–π stacking inter­actions, see: Tsuzuki *et al.* (2002 ▶).
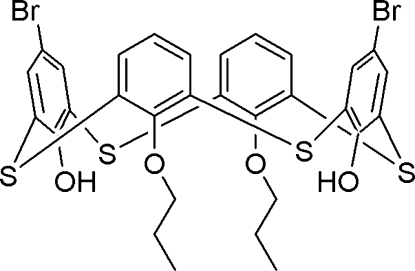


## Experimental

### 

#### Crystal data

C_30_H_26_Br_2_O_4_S_4_
*M* *_r_* = 738.57
Triclinic, 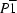

*a* = 9.3788 (16) Å
*b* = 11.712 (2) Å
*c* = 14.768 (3) Å
α = 97.904 (2)°
β = 95.614 (1)°
γ = 107.738 (2)°
*V* = 1513.5 (4) Å^3^
*Z* = 2
Mo *K*α radiation
μ = 2.99 mm^−1^
*T* = 298 K
0.29 × 0.21 × 0.20 mm

#### Data collection

Bruker SMART CCD area-detector diffractometer
Absorption correction: multi-scan (*SADABS*; Bruker, 1999 ▶) *T*_min_ = 0.478, *T*_max_ = 0.586
7993 measured reflections
5513 independent reflections
4162 reflections with *I* > 2σ(*I*)
*R*_int_ = 0.017

#### Refinement

*R*[*F* ^2^ > 2σ(*F* ^2^)] = 0.041
*wR*(*F* ^2^) = 0.108
*S* = 1.05
5513 reflections
365 parameters
H-atom parameters constrained
Δρ_max_ = 0.74 e Å^−3^
Δρ_min_ = −0.54 e Å^−3^

### 

Data collection: *SMART* (Bruker, 1999 ▶); cell refinement: *SAINT* (Bruker, 1999 ▶); data reduction: *SAINT*; program(s) used to solve structure: *SHELXS97* (Sheldrick, 2008 ▶); program(s) used to refine structure: *SHELXL97* (Sheldrick, 2008 ▶); molecular graphics: *SHELXTL* (Sheldrick, 2008 ▶); software used to prepare material for publication: *SHELXTL*.

## Supplementary Material

Crystal structure: contains datablocks I, global. DOI: 10.1107/S1600536811013043/im2275sup1.cif

Structure factors: contains datablocks I. DOI: 10.1107/S1600536811013043/im2275Isup2.hkl

Additional supplementary materials:  crystallographic information; 3D view; checkCIF report

## Figures and Tables

**Table 1:** Hydrogen-bond geometry (Å, °)

*D*—H⋯*A*	*D*—H	H⋯*A*	*D*⋯*A*	*D*—H⋯*A*
O4—H4*A*⋯O3	0.82	2.20	2.926 (3)	148
O2—H2*A*⋯O3	0.82	2.12	2.849 (3)	148

**Fig. 1.**
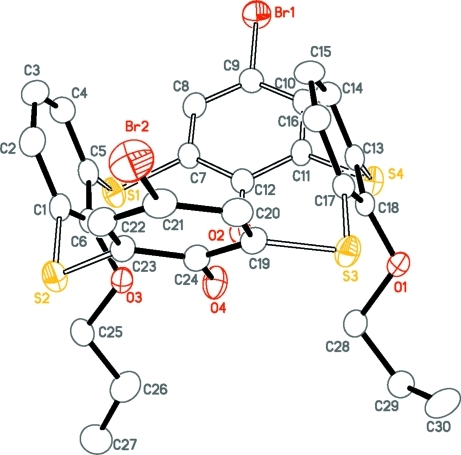
Molecular structure of the title compound with displacement ellipsoids drawn at the 30% probability level for non-H atoms. Hydrogen atoms are omitted for clarity.

**Fig. 2.**
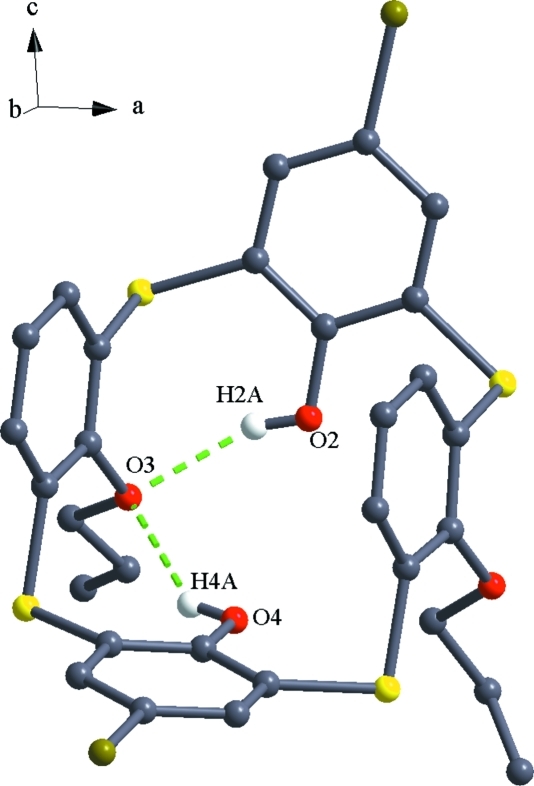
Array of intramolecular hydrogen-bonded rings of the title molecule. For the sake of clarity, H atoms not involved in hydrogen bonds have been omitted.

**Fig. 3.**
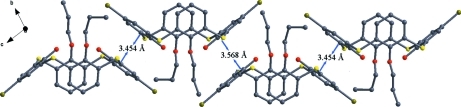
Packing diagram of the title compound, viewed along the a axis, showing offset-face-to-face π-π stacking motifs. Hydrogen atoms are omitted for clarity.

## supplementary crystallographic information

### Comment

Thiacalix[4]arenes are new versatile scaffolds for constructing highly organized receptors *via* appropriate chemical modifications at the upper or/and lower rim (Shokova & Kovalev, 2003; Lhoták, 2004; Morohashi *et al.*, 2006; Kajiwara *et al.*, 2007; Guo *et al.*, 2007). Usually, it can be attained by electrophilic bromination at the upper rim to yield the corresponding bromosubstituted thiacalix[4]arene derivatives (Lhoták *et al.*, 2001; Kasyan *et al.*, 2003; Xu *et al.*, 2008; Chen *et al.*, 2010), which can be further used to create more elaborate molecules and novel supramolecular systems. Only a few cyrstal structures of such derivatives are known, however, most of which are tetrabromothiacalix[4]arenes. Recently, Lhoták *et al.* (2001) presented the synthesis of a dibromothiacalix[4]arene, namely 5,17-dibromo-25,27-dipropoxy-26,28-dihydroxy-2,8,14,20-tetrathiacalix[4]arene, by a selective bromination reaction. We now report the crystal structure of this compound.

In the crystal structure of the title compound, as illustrated in Fig. 1, the thiacalix[4]arene unit is found in a pinched cone conformation. Two opposite ether-substituted rings, one of which is bent towards the calix cavity, are almost parallel to each other, forming a dihedral anagle of 2.99 (12)°. On the other hand, both phenolic rings are bent outwards and nearly perpendicular to each other, with an interplanar angle of 74.52 (11)° and a Br···Br distance of 13.17 (2) Å. The dihedral angles between the virtual plane defined by the four bridging S atoms and C1–C6, C7–C12, C13–C18 and C19–C24 rings are 75.04 (8), 27.12 (9), 76.01 (7) and 36.71 (10) °, respectively. Two intramolecular O—H···O hydrogen bonds (Table 1) stabilizing the cone conformation, are formed in the crystal structure. Interestingly, both OH groups make the hydrogen bonds to the same ethereal O atom, O3 (Fig. 2). A similar arrangement of such hydrogen bonds was discussed by Kasyan *et al.* (2006), while a different pattern, in which one OH group forms the hydrogen bonds to its both adjacent ethereal O atoms, was reported by Desroches *et al.* (2004).

In the packing, two different chains of molecules are formed by aromatic-aromatic interactions (Tsuzuki *et al.*, 2002). One chain, with alternating orientation, extends along the *a* axis (Fig. 3), and is established by intermolecular offset-face-to-face π-π stackings between the phenolic rings. Separations between the centroids of the phenolic rings C19–C24 and C19–C24 at (-*x*, -*y*, -*z* + 1), C7–C12 and C7–C12 at (-*x* + 1, -*y* + 1, -*z* + 2) are 3.606 (3) and 4.488 (4) Å, respectively, and the corresponding perpendicular distances are 3.454 (2) and 3.568 (2) Å. The other chain, with tail-to-tail orientation, is running along the *b* axis, with intermolecular offset-face-to-face π-π contacts between the ether-substituted rings. The distance between the centroids of the rings C1–C6 and C13–C18 at (*x* - 1, *y*, *z*) is 4.195 (2) Å, and the corresponding perpendicular distance is 3.611 (2) Å.

### Experimental

The title compound was prepared by a published procedure (Lhoták *et al.*, 2001). Single crystals of the title compound suitable for X-ray diffraction analysis were obtained by slow evaporation of a solution in CH_2_Cl_2_ and CH_3_OH (v: v = 2: 1) at 273 K.

### Refinement

All non-hydrogen atoms were refined with anisotropic displacement parameters. Hydrogen atoms attached to refined atoms were placed in geometrically idealized positions and refined using a riding model, with C—H = 0.93, 0.98 and 0.97 Å for aromatic, methylene and methyl H, respectively, and *U*_iso_(H) = 1.5Ueq(C) for methyl H, and *U*_iso_(H) =1.2Ueq(C) for all other H atoms.

### Crystal data

**Table:**

C_30_H_26_Br_2_O_4_S_4_	*Z* = 2
*M**_r_* = 738.57	*F*(000) = 744
Triclinic, *P*1	*D*_x_ = 1.621 Mg m^−^^3^
Hall symbol: -P 1	Mo *K*α radiation, λ = 0.71073 Å
*a* = 9.3788 (16) Å	Cell parameters from 2882 reflections
*b* = 11.712 (2) Å	θ = 2.5–26.7°
*c* = 14.768 (3) Å	µ = 2.99 mm^−^^1^
α = 97.904 (2)°	*T* = 298 K
β = 95.614 (1)°	Block, colourless
γ = 107.738 (2)°	0.29 × 0.21 × 0.20 mm
*V* = 1513.5 (4) Å^3^	

### Data collection

**Table:**

Bruker SMART CCD area-detector diffractometer	5513 independent reflections
Radiation source: fine-focus sealed tube	4162 reflections with *I* > 2σ(*I*)
graphite	*R*_int_ = 0.017
phi and ω scans	θ_max_ = 25.5°, θ_min_ = 1.9°
Absorption correction: multi-scan (*SADABS*; Bruker, 1999)	*h* = −10→11
*T*_min_ = 0.478, *T*_max_ = 0.586	*k* = −12→14
7993 measured reflections	*l* = −17→17

### Refinement

**Table:**

Refinement on *F*^2^	Primary atom site location: structure-invariant direct methods
Least-squares matrix: full	Secondary atom site location: difference Fourier map
*R*[*F*^2^ > 2σ(*F*^2^)] = 0.041	Hydrogen site location: inferred from neighbouring sites
*wR*(*F*^2^) = 0.108	H-atom parameters constrained
*S* = 1.05	*w* = 1/[σ^2^(*F*_o_^2^) + (0.0511*P*)^2^ + 0.5589*P*] where *P* = (*F*_o_^2^ + 2*F*_c_^2^)/3
5513 reflections	(Δ/σ)_max_ = 0.001
365 parameters	Δρ_max_ = 0.74 e Å^−^^3^
0 restraints	Δρ_min_ = −0.54 e Å^−^^3^

### Special details

**Table:**

Geometry. All e.s.d.'s (except the e.s.d. in the dihedral angle between two l.s. planes) are estimated using the full covariance matrix. The cell e.s.d.'s are taken into account individually in the estimation of e.s.d.'s in distances, angles and torsion angles; correlations between e.s.d.'s in cell parameters are only used when they are defined by crystal symmetry. An approximate (isotropic) treatment of cell e.s.d.'s is used for estimating e.s.d.'s involving l.s. planes.
Refinement. Refinement of *F*^2^ against ALL reflections. The weighted *R*-factor *wR* and goodness of fit *S* are based on *F*^2^, conventional *R*-factors *R* are based on *F*, with *F* set to zero for negative *F*^2^. The threshold expression of *F*^2^ > σ(*F*^2^) is used only for calculating *R*-factors(gt) *etc*. and is not relevant to the choice of reflections for refinement. *R*-factors based on *F*^2^ are statistically about twice as large as those based on *F*, and *R*- factors based on ALL data will be even larger.

### Fractional atomic coordinates and isotropic or equivalent isotropic displacement parameters (Å^2^)

**Table:**

	*x*	*y*	*z*	*U*_iso_*/*U*_eq_	
Br1	0.51306 (5)	0.32748 (4)	1.28607 (3)	0.06261 (15)	
Br2	−0.19390 (6)	−0.37147 (4)	0.49367 (3)	0.07472 (17)	
C1	−0.1371 (4)	0.0988 (3)	0.7904 (2)	0.0413 (8)	
C2	−0.2029 (4)	0.0044 (3)	0.8354 (3)	0.0525 (9)	
H2	−0.2691	−0.0686	0.8016	0.063*	
C3	−0.1711 (5)	0.0178 (4)	0.9301 (3)	0.0570 (10)	
H3	−0.2138	−0.0469	0.9596	0.068*	
C4	−0.0766 (4)	0.1264 (4)	0.9810 (3)	0.0526 (10)	
H4	−0.0574	0.1352	1.0450	0.063*	
C5	−0.0097 (4)	0.2227 (3)	0.9378 (2)	0.0434 (8)	
C6	−0.0376 (4)	0.2078 (3)	0.8419 (2)	0.0387 (8)	
C7	0.2766 (4)	0.3469 (3)	1.0398 (2)	0.0414 (8)	
C8	0.3184 (4)	0.3436 (3)	1.1317 (2)	0.0451 (9)	
H8	0.2522	0.3474	1.1741	0.054*	
C9	0.4581 (4)	0.3349 (3)	1.1600 (2)	0.0440 (8)	
C10	0.5564 (4)	0.3273 (3)	1.0981 (2)	0.0449 (8)	
H10	0.6499	0.3201	1.1181	0.054*	
C11	0.5164 (4)	0.3305 (3)	1.0062 (2)	0.0412 (8)	
C12	0.3760 (4)	0.3422 (3)	0.9761 (2)	0.0408 (8)	
C13	0.5253 (4)	0.1679 (3)	0.8624 (2)	0.0367 (7)	
C14	0.4575 (4)	0.0772 (3)	0.9097 (2)	0.0439 (8)	
H14	0.4753	0.0914	0.9739	0.053*	
C15	0.3638 (4)	−0.0341 (3)	0.8626 (3)	0.0505 (9)	
H15	0.3194	−0.0953	0.8948	0.061*	
C16	0.3362 (4)	−0.0543 (3)	0.7679 (2)	0.0453 (8)	
H16	0.2729	−0.1297	0.7361	0.054*	
C17	0.4008 (4)	0.0357 (3)	0.7190 (2)	0.0357 (7)	
C18	0.5011 (4)	0.1471 (3)	0.7661 (2)	0.0344 (7)	
C19	0.1502 (4)	−0.0465 (3)	0.5925 (2)	0.0387 (8)	
C20	0.0703 (4)	−0.1655 (3)	0.5527 (2)	0.0443 (8)	
H20	0.1209	−0.2168	0.5282	0.053*	
C21	−0.0847 (4)	−0.2084 (3)	0.5492 (2)	0.0467 (9)	
C22	−0.1623 (4)	−0.1353 (3)	0.5865 (2)	0.0465 (9)	
H22	−0.2667	−0.1656	0.5841	0.056*	
C23	−0.0828 (4)	−0.0161 (3)	0.6278 (2)	0.0399 (8)	
C24	0.0744 (4)	0.0297 (3)	0.6307 (2)	0.0401 (8)	
C25	−0.0263 (4)	0.3907 (3)	0.7817 (3)	0.0548 (10)	
H25A	−0.1217	0.3545	0.7407	0.066*	
H25B	−0.0457	0.4288	0.8398	0.066*	
C26	0.0777 (6)	0.4811 (4)	0.7405 (4)	0.0778 (14)	
H26A	0.0879	0.4421	0.6803	0.093*	
H26B	0.1763	0.5068	0.7784	0.093*	
C27	0.0364 (6)	0.5914 (4)	0.7288 (4)	0.0860 (16)	
H27A	−0.0710	0.5689	0.7107	0.129*	
H27B	0.0877	0.6287	0.6819	0.129*	
H27C	0.0658	0.6480	0.7861	0.129*	
C28	0.5073 (4)	0.3165 (3)	0.6889 (3)	0.0496 (9)	
H28A	0.4971	0.3710	0.7417	0.059*	
H28B	0.4071	0.2716	0.6553	0.059*	
C29	0.6031 (4)	0.3874 (3)	0.6276 (3)	0.0506 (9)	
H29A	0.7014	0.4335	0.6632	0.061*	
H29B	0.5568	0.4453	0.6080	0.061*	
C30	0.6257 (6)	0.3113 (4)	0.5436 (3)	0.0728 (13)	
H30A	0.6726	0.2542	0.5621	0.109*	
H30B	0.6895	0.3629	0.5083	0.109*	
H30C	0.5295	0.2679	0.5063	0.109*	
O1	0.5815 (3)	0.2325 (2)	0.71942 (15)	0.0412 (5)	
O2	0.3429 (3)	0.3459 (2)	0.88626 (15)	0.0491 (6)	
H2A	0.2598	0.3551	0.8768	0.074*	
O3	0.0416 (2)	0.2975 (2)	0.79765 (16)	0.0423 (5)	
O4	0.1557 (3)	0.1454 (2)	0.66895 (17)	0.0474 (6)	
H4A	0.0986	0.1822	0.6857	0.071*	
S4	0.64004 (10)	0.31392 (8)	0.92504 (6)	0.0459 (2)	
S3	0.35097 (10)	0.00864 (8)	0.59664 (6)	0.0453 (2)	
S2	−0.18282 (10)	0.08270 (9)	0.66809 (6)	0.0486 (2)	
S1	0.10033 (11)	0.36788 (9)	1.00496 (7)	0.0512 (2)	

### Atomic displacement parameters (Å^2^)

**Table:**

	*U*^11^	*U*^22^	*U*^33^	*U*^12^	*U*^13^	*U*^23^
Br1	0.0684 (3)	0.0745 (3)	0.0419 (2)	0.0225 (2)	0.00312 (19)	0.00470 (19)
Br2	0.0791 (3)	0.0426 (2)	0.0834 (3)	0.0006 (2)	0.0035 (3)	−0.0026 (2)
C1	0.0335 (18)	0.048 (2)	0.0477 (19)	0.0186 (16)	0.0124 (15)	0.0088 (16)
C2	0.045 (2)	0.047 (2)	0.068 (3)	0.0160 (18)	0.0165 (19)	0.0109 (19)
C3	0.057 (3)	0.059 (3)	0.068 (3)	0.024 (2)	0.024 (2)	0.030 (2)
C4	0.053 (2)	0.068 (3)	0.049 (2)	0.030 (2)	0.0175 (19)	0.019 (2)
C5	0.0358 (19)	0.052 (2)	0.049 (2)	0.0241 (17)	0.0109 (16)	0.0067 (17)
C6	0.0328 (18)	0.0460 (19)	0.0454 (19)	0.0214 (16)	0.0119 (15)	0.0108 (16)
C7	0.0396 (19)	0.0395 (19)	0.045 (2)	0.0158 (16)	0.0072 (16)	−0.0006 (15)
C8	0.048 (2)	0.045 (2)	0.0416 (19)	0.0154 (17)	0.0140 (17)	0.0007 (15)
C9	0.048 (2)	0.0415 (19)	0.0390 (18)	0.0144 (17)	0.0035 (16)	−0.0005 (15)
C10	0.042 (2)	0.044 (2)	0.046 (2)	0.0143 (17)	0.0018 (16)	−0.0012 (16)
C11	0.0351 (19)	0.0409 (19)	0.0448 (19)	0.0112 (15)	0.0085 (15)	−0.0008 (15)
C12	0.042 (2)	0.0375 (18)	0.0420 (19)	0.0143 (15)	0.0063 (16)	0.0016 (15)
C13	0.0290 (17)	0.0390 (18)	0.0446 (19)	0.0153 (14)	0.0079 (14)	0.0042 (15)
C14	0.048 (2)	0.049 (2)	0.0393 (18)	0.0201 (18)	0.0081 (16)	0.0118 (16)
C15	0.061 (3)	0.041 (2)	0.054 (2)	0.0167 (19)	0.0138 (19)	0.0193 (17)
C16	0.047 (2)	0.0356 (18)	0.053 (2)	0.0128 (16)	0.0079 (17)	0.0065 (16)
C17	0.0349 (18)	0.0389 (18)	0.0384 (17)	0.0178 (15)	0.0094 (14)	0.0076 (14)
C18	0.0294 (17)	0.0378 (17)	0.0409 (18)	0.0155 (14)	0.0107 (14)	0.0088 (14)
C19	0.0409 (19)	0.0436 (19)	0.0302 (16)	0.0117 (16)	0.0070 (14)	0.0051 (14)
C20	0.054 (2)	0.046 (2)	0.0348 (18)	0.0190 (18)	0.0084 (16)	0.0064 (15)
C21	0.053 (2)	0.0346 (18)	0.0427 (19)	0.0037 (17)	−0.0003 (17)	0.0039 (15)
C22	0.040 (2)	0.047 (2)	0.047 (2)	0.0054 (17)	0.0043 (16)	0.0118 (17)
C23	0.0362 (19)	0.046 (2)	0.0383 (18)	0.0118 (16)	0.0075 (15)	0.0103 (15)
C24	0.040 (2)	0.0428 (19)	0.0366 (17)	0.0113 (16)	0.0056 (15)	0.0076 (15)
C25	0.041 (2)	0.054 (2)	0.076 (3)	0.0204 (18)	0.0100 (19)	0.023 (2)
C26	0.071 (3)	0.060 (3)	0.111 (4)	0.017 (2)	0.034 (3)	0.037 (3)
C27	0.065 (3)	0.067 (3)	0.125 (4)	0.008 (2)	0.005 (3)	0.049 (3)
C28	0.047 (2)	0.048 (2)	0.064 (2)	0.0217 (18)	0.0218 (19)	0.0181 (18)
C29	0.048 (2)	0.045 (2)	0.062 (2)	0.0138 (17)	0.0131 (18)	0.0164 (18)
C30	0.093 (4)	0.072 (3)	0.064 (3)	0.027 (3)	0.038 (3)	0.028 (2)
O1	0.0362 (13)	0.0439 (13)	0.0472 (13)	0.0125 (11)	0.0154 (11)	0.0156 (11)
O2	0.0443 (15)	0.0672 (17)	0.0424 (14)	0.0265 (13)	0.0083 (11)	0.0109 (12)
O3	0.0342 (13)	0.0456 (13)	0.0503 (14)	0.0144 (11)	0.0116 (11)	0.0113 (11)
O4	0.0373 (13)	0.0400 (13)	0.0595 (15)	0.0085 (11)	0.0113 (12)	−0.0035 (11)
S4	0.0334 (5)	0.0504 (5)	0.0491 (5)	0.0100 (4)	0.0085 (4)	−0.0014 (4)
S3	0.0415 (5)	0.0544 (5)	0.0383 (5)	0.0137 (4)	0.0129 (4)	0.0011 (4)
S2	0.0390 (5)	0.0596 (6)	0.0484 (5)	0.0207 (4)	0.0022 (4)	0.0051 (4)
S1	0.0480 (6)	0.0588 (6)	0.0516 (5)	0.0303 (5)	0.0067 (4)	−0.0043 (4)

### Geometric parameters (Å, °)

**Table:**

Br1—C9	1.904 (3)	C17—S3	1.781 (3)
Br2—C21	1.893 (3)	C18—O1	1.369 (4)
C1—C2	1.382 (5)	C19—C20	1.379 (5)
C1—C6	1.395 (5)	C19—C24	1.396 (5)
C1—S2	1.786 (4)	C19—S3	1.787 (3)
C2—C3	1.377 (6)	C20—C21	1.379 (5)
C2—H2	0.9300	C20—H20	0.9300
C3—C4	1.374 (6)	C21—C22	1.376 (5)
C3—H3	0.9300	C22—C23	1.386 (5)
C4—C5	1.386 (5)	C22—H22	0.9300
C4—H4	0.9300	C23—C24	1.400 (5)
C5—C6	1.392 (5)	C23—S2	1.776 (3)
C5—S1	1.790 (4)	C24—O4	1.346 (4)
C6—O3	1.375 (4)	C25—C26	1.451 (5)
C7—C8	1.383 (5)	C25—O3	1.456 (4)
C7—C12	1.395 (5)	C25—H25A	0.9700
C7—S1	1.782 (4)	C25—H25B	0.9700
C8—C9	1.374 (5)	C26—C27	1.485 (6)
C8—H8	0.9300	C26—H26A	0.9700
C9—C10	1.373 (5)	C26—H26B	0.9700
C10—C11	1.381 (5)	C27—H27A	0.9600
C10—H10	0.9300	C27—H27B	0.9600
C11—C12	1.401 (5)	C27—H27C	0.9600
C11—S4	1.779 (3)	C28—O1	1.461 (4)
C12—O2	1.343 (4)	C28—C29	1.492 (5)
C13—C14	1.378 (5)	C28—H28A	0.9700
C13—C18	1.394 (4)	C28—H28B	0.9700
C13—S4	1.785 (3)	C29—C30	1.498 (5)
C14—C15	1.373 (5)	C29—H29A	0.9700
C14—H14	0.9300	C29—H29B	0.9700
C15—C16	1.372 (5)	C30—H30A	0.9600
C15—H15	0.9300	C30—H30B	0.9600
C16—C17	1.379 (5)	C30—H30C	0.9600
C16—H16	0.9300	O2—H2A	0.8200
C17—C18	1.395 (4)	O4—H4A	0.8200
			
C2—C1—C6	119.3 (3)	C21—C20—H20	120.0
C2—C1—S2	120.3 (3)	C19—C20—H20	120.0
C6—C1—S2	120.4 (3)	C22—C21—C20	121.3 (3)
C3—C2—C1	120.5 (4)	C22—C21—Br2	118.9 (3)
C3—C2—H2	119.8	C20—C21—Br2	119.8 (3)
C1—C2—H2	119.8	C21—C22—C23	119.2 (3)
C4—C3—C2	120.2 (4)	C21—C22—H22	120.4
C4—C3—H3	119.9	C23—C22—H22	120.4
C2—C3—H3	119.9	C22—C23—C24	120.4 (3)
C3—C4—C5	120.6 (4)	C22—C23—S2	119.6 (3)
C3—C4—H4	119.7	C24—C23—S2	119.8 (3)
C5—C4—H4	119.7	O4—C24—C19	118.4 (3)
C4—C5—C6	119.2 (3)	O4—C24—C23	122.4 (3)
C4—C5—S1	120.3 (3)	C19—C24—C23	119.2 (3)
C6—C5—S1	120.3 (3)	C26—C25—O3	108.5 (3)
O3—C6—C5	119.6 (3)	C26—C25—H25A	110.0
O3—C6—C1	120.0 (3)	O3—C25—H25A	110.0
C5—C6—C1	120.2 (3)	C26—C25—H25B	110.0
C8—C7—C12	120.3 (3)	O3—C25—H25B	110.0
C8—C7—S1	119.5 (3)	H25A—C25—H25B	108.4
C12—C7—S1	120.1 (3)	C25—C26—C27	116.5 (4)
C9—C8—C7	119.8 (3)	C25—C26—H26A	108.2
C9—C8—H8	120.1	C27—C26—H26A	108.2
C7—C8—H8	120.1	C25—C26—H26B	108.2
C10—C9—C8	121.0 (3)	C27—C26—H26B	108.2
C10—C9—Br1	119.6 (3)	H26A—C26—H26B	107.3
C8—C9—Br1	119.4 (3)	C26—C27—H27A	109.5
C9—C10—C11	120.0 (3)	C26—C27—H27B	109.5
C9—C10—H10	120.0	H27A—C27—H27B	109.5
C11—C10—H10	120.0	C26—C27—H27C	109.5
C10—C11—C12	120.1 (3)	H27A—C27—H27C	109.5
C10—C11—S4	120.0 (3)	H27B—C27—H27C	109.5
C12—C11—S4	119.9 (3)	O1—C28—C29	107.6 (3)
O2—C12—C7	123.3 (3)	O1—C28—H28A	110.2
O2—C12—C11	117.8 (3)	C29—C28—H28A	110.2
C7—C12—C11	118.9 (3)	O1—C28—H28B	110.2
C14—C13—C18	120.3 (3)	C29—C28—H28B	110.2
C14—C13—S4	119.8 (3)	H28A—C28—H28B	108.5
C18—C13—S4	119.9 (2)	C28—C29—C30	114.4 (3)
C15—C14—C13	120.5 (3)	C28—C29—H29A	108.7
C15—C14—H14	119.7	C30—C29—H29A	108.7
C13—C14—H14	119.7	C28—C29—H29B	108.7
C16—C15—C14	119.6 (3)	C30—C29—H29B	108.7
C16—C15—H15	120.2	H29A—C29—H29B	107.6
C14—C15—H15	120.2	C29—C30—H30A	109.5
C15—C16—C17	121.0 (3)	C29—C30—H30B	109.5
C15—C16—H16	119.5	H30A—C30—H30B	109.5
C17—C16—H16	119.5	C29—C30—H30C	109.5
C16—C17—C18	119.7 (3)	H30A—C30—H30C	109.5
C16—C17—S3	119.3 (3)	H30B—C30—H30C	109.5
C18—C17—S3	121.0 (2)	C18—O1—C28	116.7 (2)
O1—C18—C13	120.0 (3)	C12—O2—H2A	109.5
O1—C18—C17	121.2 (3)	C6—O3—C25	116.5 (2)
C13—C18—C17	118.7 (3)	C24—O4—H4A	109.5
C20—C19—C24	119.9 (3)	C11—S4—C13	97.33 (15)
C20—C19—S3	119.8 (3)	C17—S3—C19	97.63 (15)
C24—C19—S3	120.3 (3)	C23—S2—C1	101.49 (15)
C21—C20—C19	120.0 (3)	C7—S1—C5	103.06 (15)
			
C6—C1—C2—C3	0.2 (5)	C16—C17—C18—C13	−4.5 (5)
S2—C1—C2—C3	−178.2 (3)	S3—C17—C18—C13	174.0 (2)
C1—C2—C3—C4	1.7 (6)	C24—C19—C20—C21	1.1 (5)
C2—C3—C4—C5	−1.3 (6)	S3—C19—C20—C21	−179.9 (3)
C3—C4—C5—C6	−0.9 (5)	C19—C20—C21—C22	−1.3 (5)
C3—C4—C5—S1	174.7 (3)	C19—C20—C21—Br2	179.5 (2)
C4—C5—C6—O3	−172.5 (3)	C20—C21—C22—C23	0.4 (5)
S1—C5—C6—O3	11.9 (4)	Br2—C21—C22—C23	179.6 (3)
C4—C5—C6—C1	2.9 (5)	C21—C22—C23—C24	0.5 (5)
S1—C5—C6—C1	−172.8 (2)	C21—C22—C23—S2	175.1 (3)
C2—C1—C6—O3	172.8 (3)	C20—C19—C24—O4	−179.9 (3)
S2—C1—C6—O3	−8.8 (4)	S3—C19—C24—O4	1.1 (4)
C2—C1—C6—C5	−2.5 (5)	C20—C19—C24—C23	−0.2 (5)
S2—C1—C6—C5	175.9 (2)	S3—C19—C24—C23	−179.1 (2)
C12—C7—C8—C9	−0.5 (5)	C22—C23—C24—O4	179.1 (3)
S1—C7—C8—C9	−176.1 (3)	S2—C23—C24—O4	4.5 (5)
C7—C8—C9—C10	−1.1 (5)	C22—C23—C24—C19	−0.6 (5)
C7—C8—C9—Br1	−178.9 (3)	S2—C23—C24—C19	−175.2 (2)
C8—C9—C10—C11	1.1 (5)	O3—C25—C26—C27	−173.3 (4)
Br1—C9—C10—C11	178.9 (3)	O1—C28—C29—C30	59.9 (4)
C9—C10—C11—C12	0.4 (5)	C13—C18—O1—C28	−96.9 (3)
C9—C10—C11—S4	−177.2 (3)	C17—C18—O1—C28	86.8 (4)
C8—C7—C12—O2	−179.5 (3)	C29—C28—O1—C18	−171.2 (3)
S1—C7—C12—O2	−3.9 (5)	C5—C6—O3—C25	−91.0 (4)
C8—C7—C12—C11	1.9 (5)	C1—C6—O3—C25	93.7 (4)
S1—C7—C12—C11	177.5 (2)	C26—C25—O3—C6	176.1 (3)
C10—C11—C12—O2	179.4 (3)	C10—C11—S4—C13	111.1 (3)
S4—C11—C12—O2	−2.9 (4)	C12—C11—S4—C13	−66.5 (3)
C10—C11—C12—C7	−1.9 (5)	C14—C13—S4—C11	−44.9 (3)
S4—C11—C12—C7	175.8 (3)	C18—C13—S4—C11	133.3 (3)
C18—C13—C14—C15	−0.9 (5)	C16—C17—S3—C19	49.0 (3)
S4—C13—C14—C15	177.3 (3)	C18—C17—S3—C19	−129.5 (3)
C13—C14—C15—C16	−0.8 (5)	C20—C19—S3—C17	−114.9 (3)
C14—C15—C16—C17	−0.2 (5)	C24—C19—S3—C17	64.0 (3)
C15—C16—C17—C18	2.9 (5)	C22—C23—S2—C1	109.2 (3)
C15—C16—C17—S3	−175.6 (3)	C24—C23—S2—C1	−76.2 (3)
C14—C13—C18—O1	−172.8 (3)	C2—C1—S2—C23	−74.2 (3)
S4—C13—C18—O1	9.0 (4)	C6—C1—S2—C23	107.5 (3)
C14—C13—C18—C17	3.6 (5)	C8—C7—S1—C5	−110.4 (3)
S4—C13—C18—C17	−174.6 (2)	C12—C7—S1—C5	74.0 (3)
C16—C17—C18—O1	171.8 (3)	C4—C5—S1—C7	78.8 (3)
S3—C17—C18—O1	−9.7 (4)	C6—C5—S1—C7	−105.6 (3)

### Hydrogen-bond geometry (Å, °)

**Table:**

*D*—H···*A*	*D*—H	H···*A*	*D*···*A*	*D*—H···*A*
O4—H4A···O3	0.82	2.20	2.926 (3)	148
O2—H2A···O3	0.82	2.12	2.849 (3)	148
